# ILC2 Proliferated by IL-33 Stimulation Alleviates Acute Colitis in Rag1^−/−^ Mouse through Promoting M2 Macrophage Polarization

**DOI:** 10.1155/2020/5018975

**Published:** 2020-06-25

**Authors:** Yong You, Xiaoqing Zhang, Xiao Wang, Dan Yue, Fanxiang Meng, Junfeng Zhu, Yuanyuan Wang, Xun Sun

**Affiliations:** ^1^Department of Immunology, China Medical University, No. 77 Puhe Road, Shenyang North New Area, Shenyang, Liaoning Province, China; ^2^Laboratory Medicine Department, Sheng Jing Hospital of China Medical University, No. 36 Sanhao Street, Heping District, Shenyang, Liaoning Province, China; ^3^Department of Clinical Laboratory, Affiliated Hospital of Guilin Medical University, Guilin 541001, China; ^4^Department of Anesthesiology, The Fourth Affiliated Hospital, China Medical University, Shenyang, Liaoning Province, China

## Abstract

This study was to identify functions of ILC2, a newly found innate lymphoid cell which mainly locates in mucosa organs like lungs and intestines, in IBD. We injected rIL-33 protein to C57/BL6 mouse to explore how IL-33 induces ILC2 proliferation. The results showed that ILC2 reached a proliferation peak at day 5 and expressed multiple surface markers like CD127, C-kit, CD69, CD44, ST2, CD27, DR3, MHCII, and CD90.2. ILC2 also expressed high quantity of IL-13 and IL-5 and few IL-17A which indicates a potentially immunological function in IBD development. Afterwards, we transferred sort purified ILC2 to Rag1^−/−^ mouse given DSS to induce acute colitis in order to explore the innate function of ILC2. Data showed that ILC2 alleviates DSS-induced acute innate colitis by repairing epithelial barrier and restore body weight. Furthermore, we found that ILC2 can cause macrophages polarizing to M2 macrophages in the gut. Therefore, we concluded that ILC2 played a therapeutic role in mouse acute colitis.

## 1. Introduction

Inflammatory bowel disease (IBD) is a chronic inflammation of the intestine which primarily includes Crohn's disease and ulcerative colitis [1, 2]. It had been widely reported that genetic factors and microbiota dysregulation were primary causes of IBD [3, 4]. However, the specific pathogenesis of IBD is still not fully revealed.

IL-33, known as a member of IL-1 family [5], is described as a multiple function cytokine. IL-33 can be generated by damaged epithelial cells, adipocytes, tumors, and fibroblast [6–8]. IL-33 exerts its functions by binding to its receptor ST2 which is expressed on many immune cells like T helper cells, ILC2s, and mast cells [5, 9, 10]. It is reported that IL-33 can be involved in pathogenesis of acute colitis by promoting TH2 cell response while others believed that IL-33 played a crucial role in gut mucosa healing by inducing epithelial-derived miR-320 [11, 12]. So far, there is no overall evidence that how IL-33 functions in the intestine.

ILC2, expressing ST2, is a recently described group of immune cells which have been widely studied in its pathogenic functions in lungs but poorly understood in intestines. IL-33, IL-25, and TSLP can activate ILC2 while IL-33 alone can fully activate and maintain ILC2 [10, 13]. ILC2 expresses TH2-like cytokines, IL-4, IL-5, IL-13, and other cytokines like amphiregulin and enkephalin [14, 15].

In this study, the authors meant to determine whether and how IL-33/ILC2 axis functions in IBD.

## 2. Materials and Methods

### 2.1. Expression and Purification of rIL-33

Full-length mouse IL-33 was amplified from mouse spleen cDNA and cloned into a pUC-T vector (Beijing CoWin Biotech, Beijing, China) as previously described [16]. In brief, the insert was confirmed by direct DNA sequencing. The cDNA sequence, starting with amino acid 112 of the full-length protein, was then amplified from the above plasmid containing the mature IL-33 using specific primer pairs: 5′-GCATGAATTCATGACATTGAGCATCCAAGGAAC-3′ (forward) and 5′-CCGCCTCGAGGATTTTCGAGAGCTTAAACA-3′ (reverse). The resulting amplified fragment was inserted into the expression vector pET21a (+) at the EcoRI and XhoI to yield the construct pET21a-IL-33. This construct was transformed into *Escherichia coli* strain BL21DE3, and the expression of rIL-33 was induced by isopropyl-*β*-D-thiogalactoside and purified by using 6×His-Tagged Protein Purification Kit (Beijing CoWin Biotech, Beijing, China), followed by ToxinEraser Endotoxin Removal Kit (GenScript, Nanjing, China) to remove any endotoxin that might have come from the host cells. The purity of rIL-33 was more than 95% tested by SDS-PAGE, and the endotoxin levels were less than 1 Eu/mg of protein using Toxin Sensor Chromogenic LAL Endotoxin Assay Kit (GenScript, Nanjing, China).

### 2.2. Animals

Six-week-old male C57BL/6 mice weighing about 18-22 g were purchased from Beijing HFK Bioscience Co., Ltd. (Beijing, China). Six-week-old male Rag1^−/−^ mice on the C57BL/6 background were purchased from Animal Genetics Research Center of Nanjing University (Nanjing, China). The mice were maintained under special-pathogen-free conditions at China Medical University for at least 1 week before being used in experiments. All of the studies were performed in accordance with the China Medical University Animal Care and Use Committee guidelines.

### 2.3. Induction of Acute Colitis Model

To induce acute colitis in mice, the animals were orally administered 3% DSS (MW36000-50000, MP Biomedicals, USA) for 7 days. Mice were treated with rIL-33 (1 *μ*g in 100 *μ*L PBS/mouse/day) or PBS (100 *μ*L/mouse/day) as control on every day. Body weight and the disease activity index (DAI) were monitored daily. DAI was determined by scoring body weight loss, trait of stool, and occult blood in stool or hematochezia according to the classic scoring system described [17]. The scoring process is given as follows: body weight loss (0, none; 1, 1%–5%; 2, 5%–10%; 3, 10%–20%; 4, >20%), stool consistency (0, normal; 2, loose stool; 4, diarrhea), and stool blood (0, negative; 2, fecal occult blood test positive; 4, gross bleeding). All the animals were sacrificed on day 7, and the colon tissue and MLN were removed and cleaned then subjected to ELISA, flow cytometry, and histological analyzes.

### 2.4. Histological Assessment of Colitis

The middle part of the colon was fixed with 4% paraformaldehyde, and the fixed tissue was then embedded in paraffin. Five-micrometer tissue sections were sliced and stained with H&E. Histology was scored as described previously [11]. Histology was scored as follows: epithelium (E): 0, normal morphology; 1, loss of goblet cells; 2, loss of goblet cells in large areas; 3, loss of crypts; 4, loss of crypts in large areas and infiltration (I): 0, no infiltrate; 1, infiltrate around the crypt basis; 2, infiltrate reaching the L muscularis mucosae; 3, extensive infiltration reaching the L muscularis mucosae and thickening of the mucosa with abundant edema; 4, infiltration of the L submucosa. The total histological score was given as *E* + *I*.

### 2.5. Cell Preparation

Lamina propria (LP) cells in the colon were isolated by a modified method described previously [18]. In brief, gut pieces were cut into 2 mm slices, and the epithelium was eliminated by stirring, first in PBS containing 3 mM EDTA for 10 min at 37°C (twice) and then in RPMI (Sigma Chemical Co., St. Louis, MO, USA) containing 1% FBS, 1 mM EGTA, and 1.5 mM MgCl2 for 15 min (also twice). Gut pieces were collected and stirred in RPMI containing 20% FBS, 100 U/mL collagenase (C2139; Sigma-Aldrich Corp., St. Louis, MO, USA), and 5 U/mL DNase 1 (Sigma-Aldrich Corp) for 90 min at 37°C. Halfway through the incubation and at the end of the incubation, the suspension was dissociated by multiple aspirations through a syringe for 2 min. The pellet was purified to LPL on a 45%/66.6% discontinuous Percoll (Pharmacia, Uppsala, Sweden) gradient at 600 ×g for 20 min. MLNs from individual mice were collected under sterile conditions in ice-cold PBS with 10% fetal calf serum (FBS). Then, the lymph nodes were gently disrupted with a sterile syringe plunger and filtered through a nylon cell strainer (40 *μ*m mesh; BD Biosciences, San Jose, CA, USA). The cells were collected after centrifugation at 1500 rpm at room temperature for 5 min. The number of viable cells was counted by trypan blue staining.

### 2.6. Enzyme-Linked Immunosorbent Assay (ELISA)

To measure spontaneous cytokines produce by LPL. Isolated LPL were measured with ELISA kits (R&D Systems) including IFN-*γ*, IL-17A, IL-4, IL-10, IL-5, and IL-13 according to the manufacturer's instruction.

### 2.7. Flow Cytometry

Isolated LPL were incubated with an FcgR-blocking mAb and stained with mAbs against mouse CD3, B220, CD25, C-kit, CD27, CD69, CD44, ST2, CD127, I-A/I-E, DR3, CD90.2, NK1.1, CD11b, CD11c, NKp64, and CD206 (Biolegend, San Diego, CA 92121). For intracellular cytokine staining, LPL or MLN were stimulated with PMA (25 ng/mL; Sigma-Aldrich, St. Louis, MO) and ionomycin (1 *μ*g/mL; Sigma-Aldrich) for 5 h at 37°C. Brefeldin A (10 *μ*g/mL; Sigma-Aldrich) was added after the first hour of incubation. These cells were harvested, washed, and stained with mAbs against mouse IL-13, IL-4, IL-10, IL-5, IL-17A, or IFN-*γ* for 30 min at 4°C. The intracellular expression of IL-13, IL-4, IL-10, IL-5, IL-17A, or IFN-*γ* in LPL or MLN was analyzed by flow cytometry using a Cytofix/Cytoperm Kit Plus (BD Biosciences, San Jose, CA) according to the manufacturer's instructions. For ILC2 cell sorting, a BD FACSAriaII (BD bioscience, U.S.A) was used to purify ILC2s. Cells from spleen were stained with CD3, B220, CD25, and ST2. ILC2s were identified as CD3^−^B220^−^CD25^+^ST2^+^ cells. The purity of ILC2 population was 95%, as verified by postsort flow cytometric analysis.

### 2.8. Adoptive Transfer Assays

Sorted purified ILC2s (CD3^−^B220^−^CD25^+^ST2^+^) were isolated from spleens of male C57BL/6 mice. ILC2s were adoptively transferred into Rag1^−/−^mice. After 7 days, transferred mice were analyzed or treated with 3% DSS for indicated days for further assays.

### 2.9. Statistical Analysis

The difference in survival rates was assessed by the log rank test (Mantel-Cox). Differences in parametric data were evaluated by a Student's *t*-test. Statistically significant differences were accepted when *p* < 0.05.

## 3. Results and Discussion

### 3.1. IL-33 Induces ILC2 Proliferating in MLN and LPL

**Figure 1:**
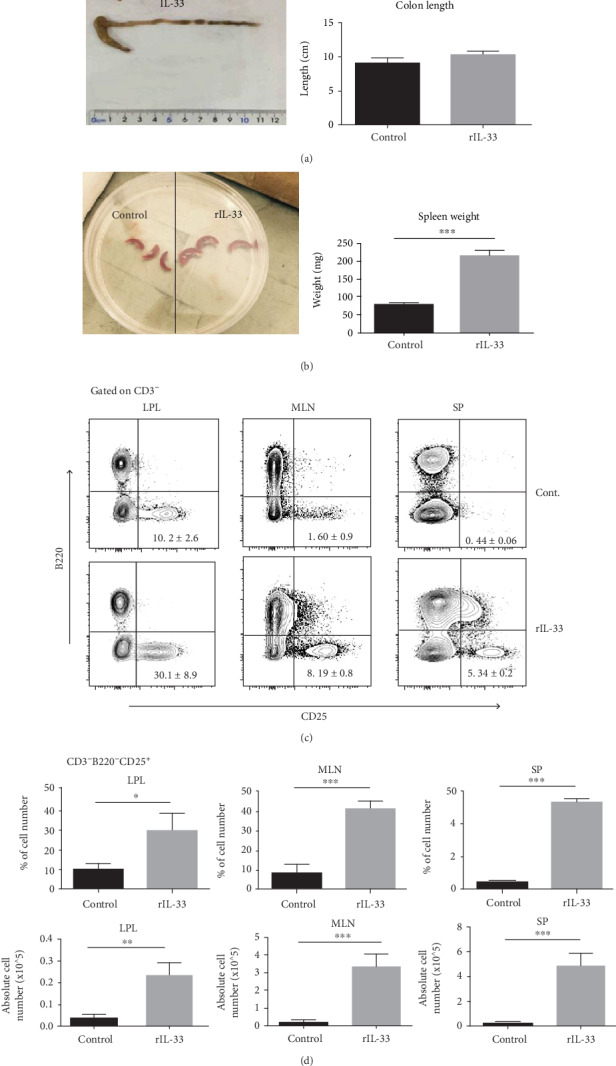
IL-33 intraperitoneal injected to WT C57/BL6 male mouse while control group was administrated by PBS. Lymphocyte subsets of the LPL or MLN of IL-33- and PBS-treated mice were analyzed by flow cytometry. (a) Colon length and (b) spleen weight were analyzed on day 7. (c) The frequency of CD3-B220-CD25+ cells was isolated from the LPL or MLN on day 7. The absolute numbers of cells were counted on the same day. Data indicate mean ± SD of each group (*n* = 5/group) obtained from a representative of three independent experiments. Statistically significant differences are shown (^∗^*p* < 0 05, ^∗∗^*p* < 0 01, or ^∗∗∗^*p* < 0 001).

For starters, we investigated IL-33 functions by intraperitoneal injecting 10 *μ*g/mL IL-33 for 7 days with C57BL/6 mouse. Flow cytometry results showed IL-33 significantly enhanced CD3^−^B220^−^CD25^+^ cells both in absolute number and percentage in LPL, MLN, and spleen (Figures 1(c) and 1(d)). Moreover, compared with the different organs in the control group, we found that these CD3^−^B220^−^CD25^+^ cells tend to locate in the intestine which presents similar feature as ILC2 tends to locate in mucosa organs. Therefore, we primarily assumed that CD3^−^B220^−^CD25^+^ cells enhanced by IL-33 were ILC2s.

To avoid confounding factors, we check whether intraperitoneal injecting IL-33 may cause some sort of inflammation in the guts (Figure 1(a)). Data showed that after a dose of 10 *μ*g/mL rIL-33 injection, mouse showed no sign of suffering colitis. However, the enhanced spleen weight of these mice showed a systemic effect of IL-33 (Figure 1(b)).

### 3.2. IL-33-Derived CD3^−^B220^−^CD25^+^ Cells Were ILC2s

**Figure 2:**
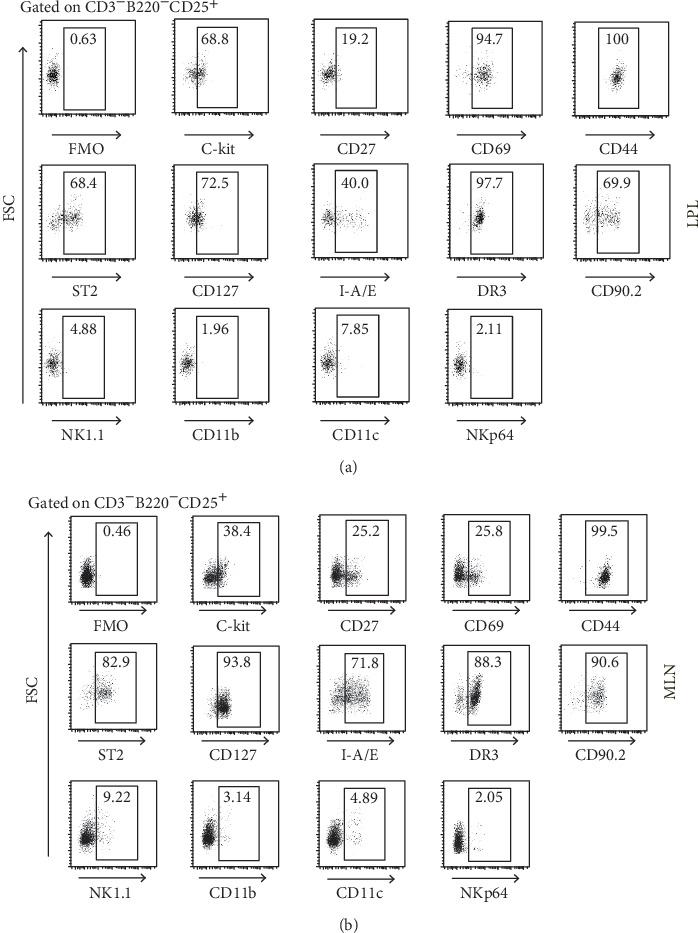
IL-33 intraperitoneal injected to WT C57/BL6 male mouse. Lymphocyte subsets of the LPL or MLN of IL-33-treated mice were analyzed by flow cytometry. Phenotype (C-kit, CD27, CD69, CD44, ST2, CD127, I-A/I-E, DR3, CD90.2, NK1.1, CD11b, CD11c, and NKp64) of CD3-B220-CD25+ cells (a) in LPL and (b) in MLN.

Afterwards, we examined surface marker that CD3^−^B220^−^CD25^+^ cells might express. As we previously thought, CD3^−^B220^−^CD25^+^ cells expressed C-kit, CD127, ST2, and DR3 but not NK1.1, CD11b, CD11c, and NKp64 (Figure 2). These data proved our former assumption that these CD3^−^B220^−^CD25^+^ cells induced by IL-33 were ILC2s.

### 3.3. A Time Course Showed ILC2 Proliferated the Most on Day 5

**Figure 3:**
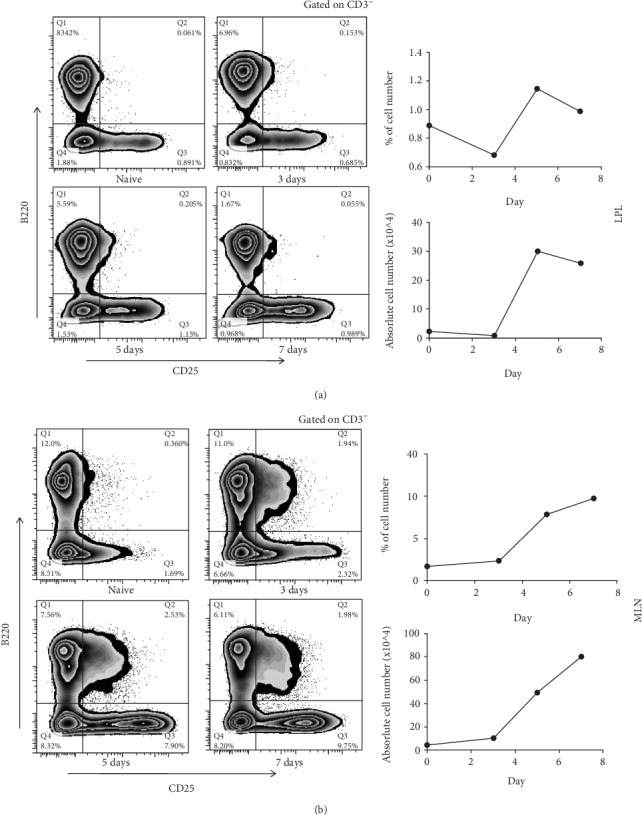
IL-33 intraperitoneal injected to WT C57/BL6 male mouse. CD3-B220-CD25+ cells of the LPL or MLN of IL-33-treated mice were analyzed by flow cytometry on defaulted days. Cell number and percentage change (a) in LPL and (b) in MLN.

In order to perform further investigation, we examined how ILC2 proliferates in a time manner. It turns out that ILC2 reached its proliferation peak on day 5 (Figure 3(a)) while it continued proliferating in MLN. Thus, we decided that our further experiment will be performed on day 5 (Figure 3(b)).

### 3.4. ILC2 Expresses Abundant TH2-like Cytokine

**Figure 4:**
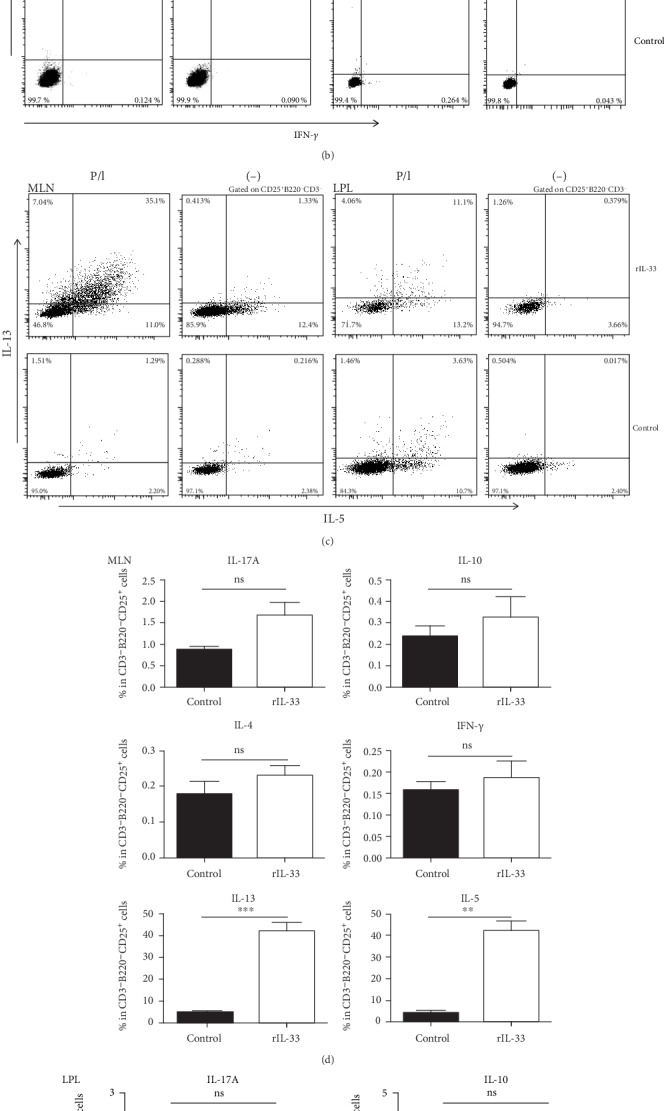
The population of cytokine-producing ILC2 in the MLN or LPL of IL-33- and PBS-treated mice was analyzed by flow cytometry. (a–c) The frequencies of IL-17A+IL-10+IL-4+IFN-*γ*+IL-5+IL-13+ILC2 in MLN and LPL of IL-33-treated mice with or without P/I stimulation. (d, e) The frequencies of cytokine expressions ILC2 in MLN and LPL of IL-33-treated mice with or without P/I stimulation. Data indicate mean ± SD of each group (*n* = 5/group) obtained from a representative of three independent experiments (^∗^*p* < 0 05, ^∗∗^*p* < 0 01, ^∗∗∗^*p* < 0 001).

To determine potential function of ILC2, we examined cytokine expression by performing intracellular staining and analyzing with flow cytometry. ILC2 expressed abundant IL-13 and IL-5, slightly IL-4 and IL-17, and almost no sign of IL-10 and IFN-*γ*. Moreover, comparing LPL to MLN in control group (Figures 4(a)–4(e)), ILC2 expressed more IL-13 and IL-5 which means ILC2 can be activated without any external stimuli in the gut. Thus, we believed that ILC2 in the gut may play an important role.

### 3.5. ILC2 Plays a Therapeutic Role in DSS-Induced Acute Colitis in Rag1^−/−^ Mouse

**Figure 5:**
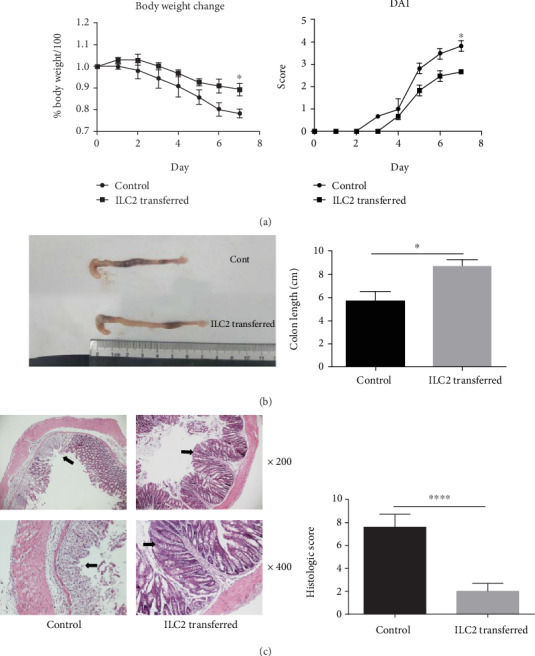
ILC2 adaptive transfer treatment attenuates DSS-induced acute colitis in the Rag1^−/−^ mice. Mice were orally treated with 3% DSS in the drinking water to induce colitis as described in Materials and Methods. (a) Body weight and disease activity index were daily observed. On day 7, (b) macroscopic changes, colon length, and (c) histological score (magnification, ×200 and ×400) were analyzed. Data indicate mean ± SD of each group (*n* = 3/group) obtained from a representative of three independent experiments (^∗^*p* < 0 05, ^∗∗^*p* < 0 01, ^∗∗∗∗^*p* < 0 0001).

Due to the abundant TH2-like cytokine ILC2 expressed, Rag1^−/−^ mouse was employed to eliminate potential effect of T and B cells in the gut to make our point more specific. We transferred sort-purified ILC2 to Rag1^−/−^ mouse which afterwards administrated by 5 days DSS to induce an acute colitis. DAI and H&E staining were used to evaluate severity of this innate colitis. Data showed that Rag1^−/−^ mouse transferred with ILC2 presents a significant decrease in histological score and DAI (Figures 5(a) and 5(b)). According to H&E staining, epithelial and most part of lamina propria were damaged in control group while they are protected in ILC2-transferred group (Figure 5(c)).

### 3.6. ILC2 Upregulates IL-13 and IL-5 Level and Causes M2 Macrophages Polarization in LPL

**Figure 6:**
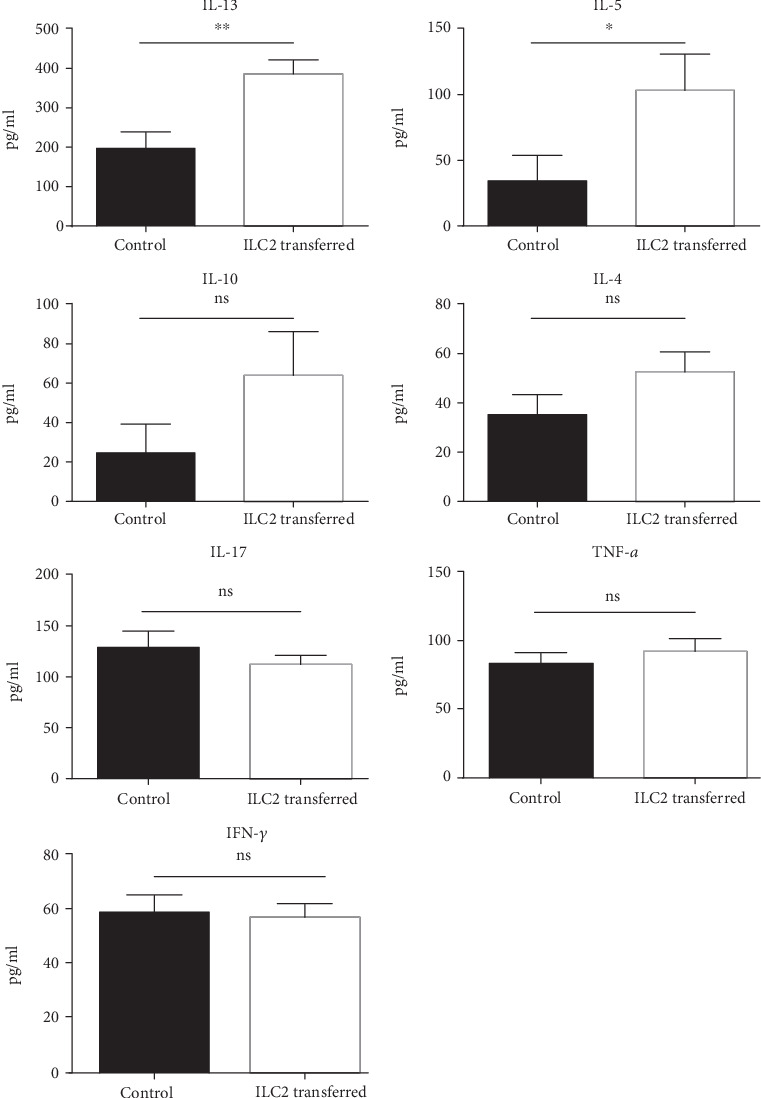
Cytokine production by lymphocytes isolated from the LPL: ILC2 adaptive transferred and PBS-treated mice with DSS-induced colitis. LPL cells measured by ELISA assay. Data indicate mean ± SD of each group (*n* = 3/group) obtained from a representative of three independent experiments and were valued by a Student's *t*-test (^∗^*p* < 0 05, ^∗∗^*p* < 0 01).

**Figure 7:**
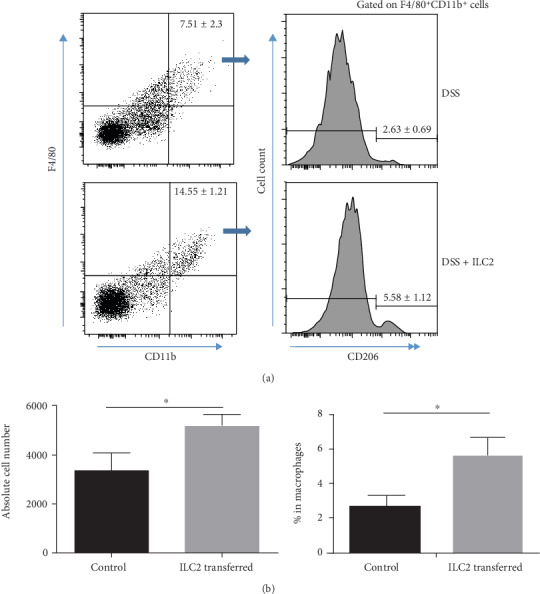
ILC2 adaptive transfer treatment attenuates DSS-induced acute colitis in the Rag1^−/−^ mice. Mice were orally treated with 3% DSS in the drinking water to induce colitis as described in Materials and Methods. M1 macrophage (F4/80+CD11b+CD206-) and M2 macrophage (F4/80+CD11b+CD206+) were measured by flow cytometry. (a) Percentage of macrophage and CD206 expression. (b) Absolute cell number and frequencies of M2 macrophage. Data indicate mean ± SD of each group (*n* = 3/group) obtained from a representative of three independent experiments and were valued by Student's *t*-test (^∗^*p* < 0 05).

After ILC2 treatment, IL-13 and IL-5 were remarkably upregulated while other IBD-associated cytokines did not show obvious change (Figure 6(a)). Furthermore, we examined potential effect on several innate lymphocytes. Data showed an increase in cell number of macrophages (Figure 7(a)). Afterwards, we examined possible macrophage polarization behavior. Those mice treated with ILC2 showed an increase in CD206^+^ M2 macrophage in cell number (Figures 7(a) and 7(b)). These results provide evidence that ILC2 plays a therapeutic role in mice DSS-induced acute colitis.

## 4. Discussion

IL-33 as an alarmin was widely discussed about its functions in several different types of autoimmune diseases [19, 20]. Some researches show that IL-33 was a proinflammatory factor [16, 21], while others prove that it provokes a remedy mechanism [22–24]. In our study, we first explored how rIL-33 affects immune cells in vivo. And as many authors had proved, IL-33 induced ILC2 proliferation. Considering that ILC2s were mucosal organs resident cells, we decided to further investigate how ILC2 functions in intestines.

IL-33 plays a complicated function in IBD. Zhu et al. [11] showed that IL-33 aggravates DSS-induced chronic colitis by enhancing Th2 cell response while he showed a different outcome in DSS-induced acute colitis [22]. To better understand this contradiction, we thought to concentrate on the effect of innate lymphocyte. Our data showed that after IL-33 administration, ILC2 starts to proliferate instantly at day 1 and reached its peak at day 5 (Figures 1(c), 3(a), and 3(b)). At the meantime, ILC2 released a large amount of TH2-like cytokine like IL-5 and IL-13 (Figures 4(a)–4(e)) which supported many researches that showed an upregulation of Th2 cell respond in acute colitis after IL-33 administrate [25, 26]. When it comes to chronic colitis, some researchers believed that IL-33 alleviates the syndrome by suppressing Th17 and Th1 responses [27]. Indeed, ILC2 secreted TH2-like cytokine can balance Th1 response. However, no evidence showed that Th2-like cytokine can suppress Th17 response. Thus, we assume there might be other mechanisms in chronic colitis.

Our data showed that ILC2 can promote macrophage polarization which leads to an increase in M2 macrophage number (Figure 6(b)). Interestingly, Zhou et al. [28] found that IL-13 treatment could inhibit YAP expression via the PI3K-AKT-*β*-catenin pathway, while YAP can suppress macrophages polarize to M2 macrophages which leads to aggravating colitis [29]. Therefore, we believed that IL-13 could alleviate colitis by suppressing YAP function to enhance M2 macrophage polarization. In our study, ILC2 expresses large amount of IL-13 (Figures 4(c) and 4(d)) and causes an observable increase in LPL (Figure 6(a)). It suggests that ILC2 plays its therapeutic role by upregulating IL-13 to promote M2 macrophage polarization.

Other evidence shows that ILC2 could express AREG in MLN which can upregulate downstream EGFR level and ultimately result in intestinal barrier repair [17, 30]. This mechanism could be a fact that suggests how ILC2 alleviates colitis in mouse. However, Hardbower et al. [31] demonstrated that EGFR signal in the gut could lead to tumorigenesis by suppressing both M1 and M2 macrophages. Together with our data, we believe that the therapy targeting on IL-33/ILC2/M2-macrophage axis could be a more effective and safer way.

## 5. Conclusion

Taken together, our study suggests that IL-33 proliferating ILC2 can exert a therapeutic role in mouse colitis. ILC2 also promotes M2 macrophage polarization by secreting high level of IL-13 in vivo which could attenuate intestine inflammation.

## Data Availability

The data used to support the findings of this study are available from the corresponding author upon request.

## Abbreviations

ILC2: Group 2 innate lymphoid cells
DSS: Dextran sulfate salt
IBD: Inflammatory bowel disease
IL-33: Interleukin33
rIL-33: Recombinant interleukin33
LPL: Lamina propria lymphocytes
H&E: Hematoxylin and eosin
MLN: Mesenteric lymph nodes
Cont: Control
SP: Spleen
TH2: T helper 2 cell
AREG: Amphiregulin
EGFR: Epithelial growth factor receptor.
