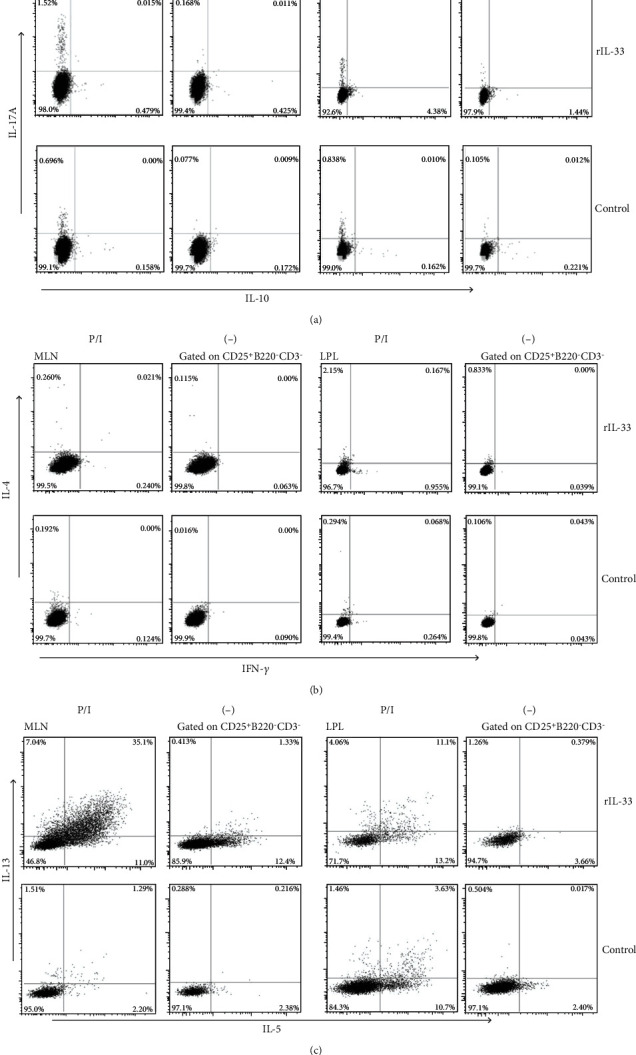# Erratum to “ILC2 Proliferated by IL-33 Stimulation Alleviates Acute Colitis in Rag1^−/−^ Mouse through Promoting M2 Macrophage Polarization”

**DOI:** 10.1155/2021/9843489

**Published:** 2021-05-11

**Authors:** Yong You, Xiaoqing Zhang, Xiao Wang, Dan Yue, Fanxiang Meng, Junfeng Zhu, Yuanyuan Wang, Xun Sun

**Affiliations:** ^1^Department of Immunology, China Medical University, No. 77 Puhe Road, Shenyang North New Area, Shenyang, Liaoning Province, China; ^2^Laboratory Medicine Department, Sheng Jing Hospital of China Medical University, No. 36 Sanhao Street, Heping District, Shenyang, Liaoning Province, China; ^3^Department of Clinical Laboratory, Affiliated Hospital of Guilin Medical University, Guilin 541001, China; ^4^Department of Anesthesiology, The Fourth Affiliated Hospital, China Medical University, Shenyang, Liaoning Province, China

In the article titled “ILC2 Proliferated by IL-33 Stimulation Alleviates Acute Colitis in Rag1^−/−^Mouse through Promoting M2 Macrophage Polarization” [1], Figures 4(a)–4(c) are mistakenly duplicated due to an error in the production process. The correct Figure 4 is as follows:

## Figures and Tables

**Figure 1:**